# Postoperative effect of sufentanil preemptive analgesia combined with psychological intervention on breast cancer patients

**DOI:** 10.1186/s12871-023-02143-8

**Published:** 2023-05-20

**Authors:** Hong Tan, Chengqiang Wang, Yihong Jiang, Quan Shi, Wei Liang, Di Li

**Affiliations:** 1 Department of Anesthesiology, the Affiliated Hospital of Guilin Medical University, Guilin, 541001 Guangxi China; 2 Department of Epidemiology and Statistics, School of Public Health, Guilin Medical University, Guilin, 541199 Guangxi China

**Keywords:** Sufentanil, Preoperative analgesia, Psychological intervention, Breast cancer patients

## Abstract

**Objectives:**

To explore the postoperative effects of sufentanil preemptive analgesia combined with psychological intervention on breast cancer patients undergoing radical surgery.

**Methods:**

112 female breast cancer patients aged 18–80 years old who underwent radical surgery by the same surgeon were randomly divided into 4 groups, and there were 28 patients in each group. Patients in group A were given 10 µg sufentanil preemptive analgesia combined with perioperative psychological support therapy (PPST), group B had only 10 µg sufentanil preemptive analgesia, group C had only PPST, and group D were under general anesthesia with conventional intubation. Visual analogue scoring (VAS) was used for analgesic evaluation at 2, 12 and 24 h after surgery and compared among the four groups by ANOVA method.

**Results:**

The awakening time of patients in group A or B was significantly shorter than that in group C or D, and the awakening time in group C was significantly shorter than that in group D. Moreover, patients in group A had the shortest extubation time, while the group D had the longest extubation time. The VAS scores at different time points showed significant difference, and the VAS scores at 12 and 24 h were significantly lower than those at 2 h (*P* < 0.05). The VAS scores and the changing trend of VAS scores were varied among the four groups (*P* < 0.05). In addition, we also found that patients in group A had the longest time to use the first pain medication after surgery, while patients in group D had the shortest time. But the adverse reactions among the four groups showed no difference.

**Conclusions:**

Sufentanil preemptive analgesia combined with psychological intervention can effectively relieve the postoperative pain of breast cancer patients.

## Background

Breast cancer is the most prevalent malignancy in women worldwide, accounting for approximately 25% of all kinds of cancers [1]. The latest cancer reports show that breast cancer is still in the first place among female malignancies, with new cases accounting for 30% of all cancers, and the mortality rate is second only to lung cancer, at about 15% [2]. Early detection and early treatment are important for reducing the mortality of breast cancer and improving the quality of life of breast cancer patients.

The use of radical surgery can help the majority of breast cancer patients. Due to the extensive resection of radical surgery, breast cancer patients always feel pain after surgery [3]. There are about 25–60% of breast cancer patients affected by persistent postoperative pain after breast cancer treatment [4, 5]. Due to persistent postoperative pain of breast cancer patients can affect the upper limb function and activities of daily living, it is important to reduce the persistent postoperative pain for breast cancer survivors [6]. For this reason, researchers have a special interest in breast cancer anesthesia and its postoperative analgesia. Some studies have found that preemptive analgesia can improve the analgesia effect and reduce inflammatory response of breast cancer patients [7, 8]. Sufentanil is a highly selective µ-opioid agonist with few side effects and experienced clinical use [9, 10]. Preemptive analgesia with sufentanil shows effectiveness in reducing postoperative pain in patients [11, 12].

As the progression of breast cancer is completely unpredictable, the anxiety and worry of breast cancer patients is inevitable, which may be worry, anxiety, fear, impatience or irritability. Studies have shown that breast cancer patients face not only the stress of surgery, but also post-operative physical defects and a range of negative emotions due to the uncertainty of disease progression and healing [13, 14]. There are several studies on psychological interventions to help patients have been reported. For example, psychological interventions have significant clinical efficacy on recovery and improvement of life quality in esophageal cancer patients [15]. Hadlandsmyth et al. reported a single psychological intervention for women undergoing breast cancer surgery and found that it posed some small positive effects on postsurgical pain and anxiety [16]. Positive psychological intervention based on PERMA model is found to have a significant effect on perioperative patients with AIDS complicated with breast cancer [17]. The postoperative anxiety, depression, fatigue, and anger of breast cancer patients can be significantly improved by psychological intervention-assisted comfort nursing after breast cancer surgery [18].

With the development of anesthesiology and the introduction of the concept of comfort care, comprehensive research in clinical anesthesia has received increasing attention from a wide range of scholars [19–22]. From the above literatures, it is clear that preemptive analgesia and psychological interventions have better results for surgical patients, respectively. However, the combined analgesic effect of preemptive analgesia and preoperative psychological intervention on breast cancer patients undergoing radical surgery still has not been explored.

At present, sufentanil has been widely used in postoperative analgesia, but there are few studies in preemptive analgesia, and breast cancer patients also need more psychological support. In this study, sufentanil preemptive analgesia combined with psychological support therapy were used to explore the postoperative effects on breast cancer patients undergoing radical surgery. We aim to provide a safe and effective anesthetic method for comprehensive postoperative pain relief in breast cancer patients.

## Methods

### Patients and grouping

One hundred and twelve female patients who voluntarily requested breast cancer surgery were selected from the Affiliated Hospital of Guilin Medical University between January 2015 and January 2017. The surgery of all patients was performed by the same surgeon. All patients were informed with the study and wrote the consent. This study was approved by the Ethical Boards of the Affiliated Hospital of Guilin Medical University and the registration number is 2022QTLL-09.

Inclusion criteria for patient selecting: (1) Underwent unilateral radical surgery of breast cancer; (2) Had no metastasis of breast cancer; (3) Aged 18–80 years old; (4) At I-II level of American Society of Anesthesiologists Classification.

Exclusion criteria for patient selecting: (1) Had hypertension, diabetes, cardiopulmonary, hepatic or renal diseases, or disorders of water-electrolyte balance; (2) Had history of relevant drug allergies, history of opioid or alcohol abuse, history of taking antidepressants and psychological drugs, or history of chronic pain; (3) Had addiction to opiates or psychedelic drugs; (4) Had history of taking analgesics and NSAIDs in the 48 h before surgery; (5) Had speech, hearing, vision or other severe psychological disorders that do not cooperate with perioperative psychological support therapy.

The patients were divided into four groups and there were 28 patients in each group. Patients in group A were given sufentanil preemptive analgesia combined with psychological intervention, patients in group B had only sufentanil preemptive analgesia, patients in group C had only perioperative psychological support therapy (PPST), and patients in group D were under general anesthesia with conventional intubation.

### Perioperative psychological support therapy (PPST)

At the day before the surgery, face-to-face communication and routine visits were given to allow patients sign the informed consent for anesthesia and get psychological interventions. The exact procedures were as follows:

1) Cognitive input: giving photos of people wearing surgical gowns and scenes related to work in the operating room, introducing the basic process of entering the operating room on the day of surgery and the basic steps of general anesthesia; helping patients to have a correct perception of anesthesia with the help of popular science articles as well as videos and pictures.

2) Anxiety self-assessment scale: to understand their level of stress and anxiety, the reasons, and individualized analysis to help.

3) Cue therapy: confident and assertive attitude of the anesthesiologist informing about the method of anesthesia, contingency plans, and safety.

4) Successful case desensitization: using the cases of successful postoperative patients to remove patients’ fears and increase their confidence.

5) Postoperative follow-up on analgesic satisfaction and related psychological conditions of breast cancer patients after surgery.

### Analgesia methods

Group A: Anesthesiologists explained the purpose of tracheal intubation to patients 30 min before surgery, showed and acquainted patients with the tracheal tube, and instructed patients in advance to open their mouths to simulate postoperative cooperation with tracheal extubation. Then the patients were given 10 µg sufentanil (diluted to 10 mL with saline) by intravenous injection, and PPST by professional staff according to standard procedures.

Group B: Patients were given 10 µg of sufentanil (diluted to 10 mL with saline) intravenously 30 min before surgery.

Group C: Patients were explained the purpose of tracheal intubation 30 min before surgery, shown and acquainted with the tracheal tube, and instructed in advance to open their mouths to simulate postoperative cooperation with tracheal extubation, followed by intravenous injection of 10 mL saline.

Group D: Patients received conventional intubation with general anesthesia, without any preemptive analgesia or psychological support treatment.

### Anesthesia methods

All breast cancer patients were intubated with general anesthesia, routinely abstained from drinking for 6 h and from eating for 8 h before surgery. One vein was opened after the patient was admitted to the room. Before the start of the surgery, all patients were intravenously injected with 0.07 mg/kg midazolam, 0.2 mg/kg etomidate, 0.5 µg/kg sufentanil and 0.7 mg/kg rocuronium. After entering anesthesia, patients were maintained by sedation with 4–10 mg/(kg•h) propofol + 1% sevoflurane, analgesia with 0.05–0.25 µg/(kg•min) remifentanil, muscle relaxation with 0.3–0.6 mg/kg rocuronium maintenance, and muscle relaxants were stopped 35 min before the end of surgery. The target-controlled concentration of propofol, remifentanil and rocuronium was adjusted at any time during the operation according to the patient’s body movement response, the change of heart rhythm, and blood pressure until the end of the operation. Propofol and remifentanil were stopped at the end of the operation in all four groups, and 20 µg/kg neostigmine and 10 µg/kg atropine were administered to antagonize the residual effect of muscle relaxants. All patients were given 3 mg granisetron IV at the end of surgery and appropriate intraoperative fluid rehydration according to the situation, and the tracheal tube was removed after the patients had the conditions for extubation at the end of surgery.

### Analgesic evaluation

Visual Analogue Scale/Score (VAS) is used for analgesic evaluation. VAS is divided into a linear graph with a 10 cm × 3 cm linear scale, where “0” on the left side indicates no pain and “10” on the right side indicates the most severe pain imaginable. The pain scores of the four groups were compared at 2, 12 and 24 h after surgery. The time of surgery, time of awakening and time of extubation were recorded for the four groups. The patients were observed for complications such as respiratory depression, cardiac arrhythmia, and gastrointestinal bleeding. The use of other analgesics on demand, and the occurrence of nausea, vomiting, sleepiness and pruritus were recorded in the 24 h postoperative period.

### Statistical analyses

G*Power 3.1.9.7 software was used for sample size calculation. According to our pre-experiment, the effect size was set at 0.4 which is a large Cohen’s univariate effect size convention, the test level was set at 0.05, and the test power was 0.90. The total sample size was 93. Considering the loss to follow-up, 112 patients were collected. SPSS 23.0 was applied to analyze all the data. Mean ± standard deviation (SD) was used for the quantitative data. One-way ANOVA was used for the comparison of the four groups, SNK test was used for the comparison between two groups, and repeated-measures ANOVA was used for the repeated-measures data. For the qualitative data, chi-square test was used for the comparison between groups. Differences were considered statistically significant at *P* < 0.05.

## Results

### Group comparison of general information

**Fig. 1:**
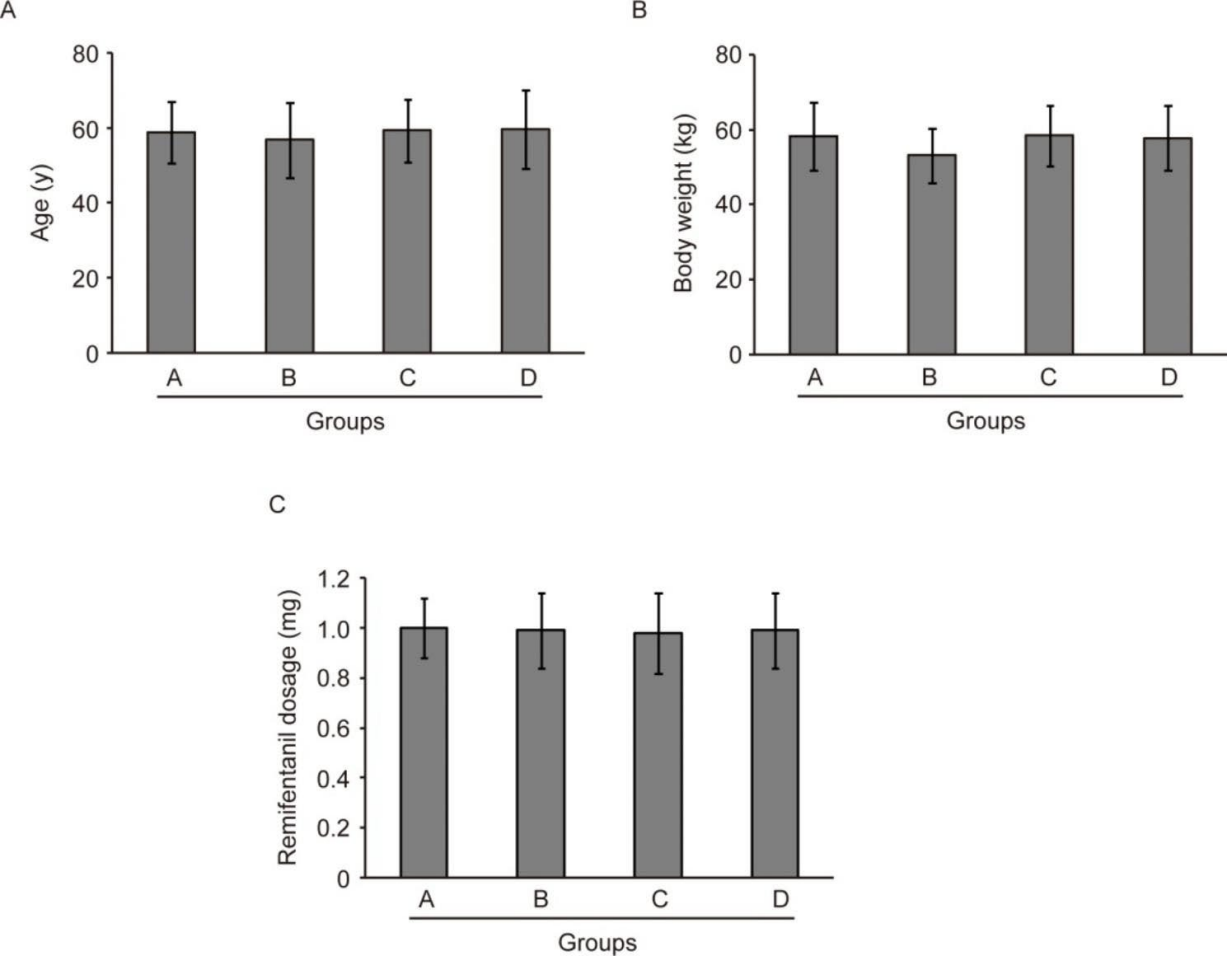
Comparison of age (**A**), body weight (**B**), and remifentanil dosage (**C**) of breast cancer patients in the four groups

The general information of the 112 breast cancer patients, such as age, weight, and intraoperative remifentanil dosage, was collected and compared among the four groups. As shown in Fig. 1, there were no statistically significant differences of age, weight, or intraoperative remifentanil dosage (all *P* > 0.05), indicating that the four groups had a comparable baseline.

### Group comparison of surgery time, awakening time and extubation time

**Fig. 2:**
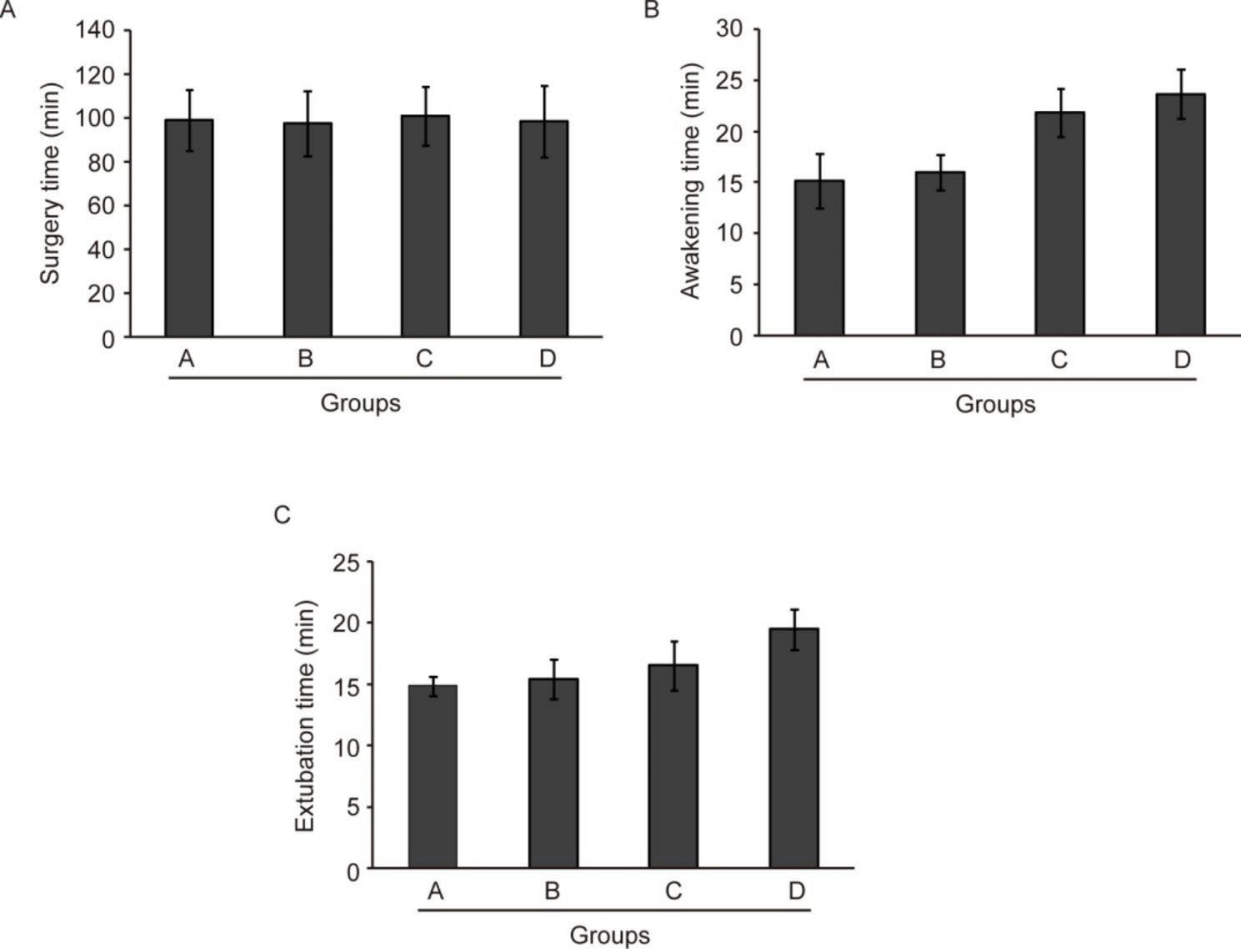
Comparison of surgery time (**A**), awakening time (**B**), and extubation time (**C**) of breast cancer patients in the four groups undergoing surgery

The surgery time of patients in the four groups was first compared and the result showed no statistically significant differences (Fig. 2A, *P* > 0.05). However, there was a statistically significant difference of the awakening time among the four groups by one-way ANOVA analysis (Fig. 2B, F = 95.217, *P* < 0.05). Further SNK test showed that the awakening time of patients in group A or group B was significantly shorter than that in groups C or D, and the awakening time of patients in group C was significantly shorter than that in group D (all *P* < 0.05). Moreover, the extubation time of patients in the four groups was also significantly different (Fig. 2C, F = 95.217, *P*<0.05). The breast cancer patients in group A had the shortest extubation time, while the group D had the longest extubation time (all *P* < 0.05). These results show that sufentanil preemptive analgesia combined with psychological intervention can decrease both the awakening time and extubation time of breast cancer patients after surgery.

### Group comparison of analgesic effects

**Fig. 3:**
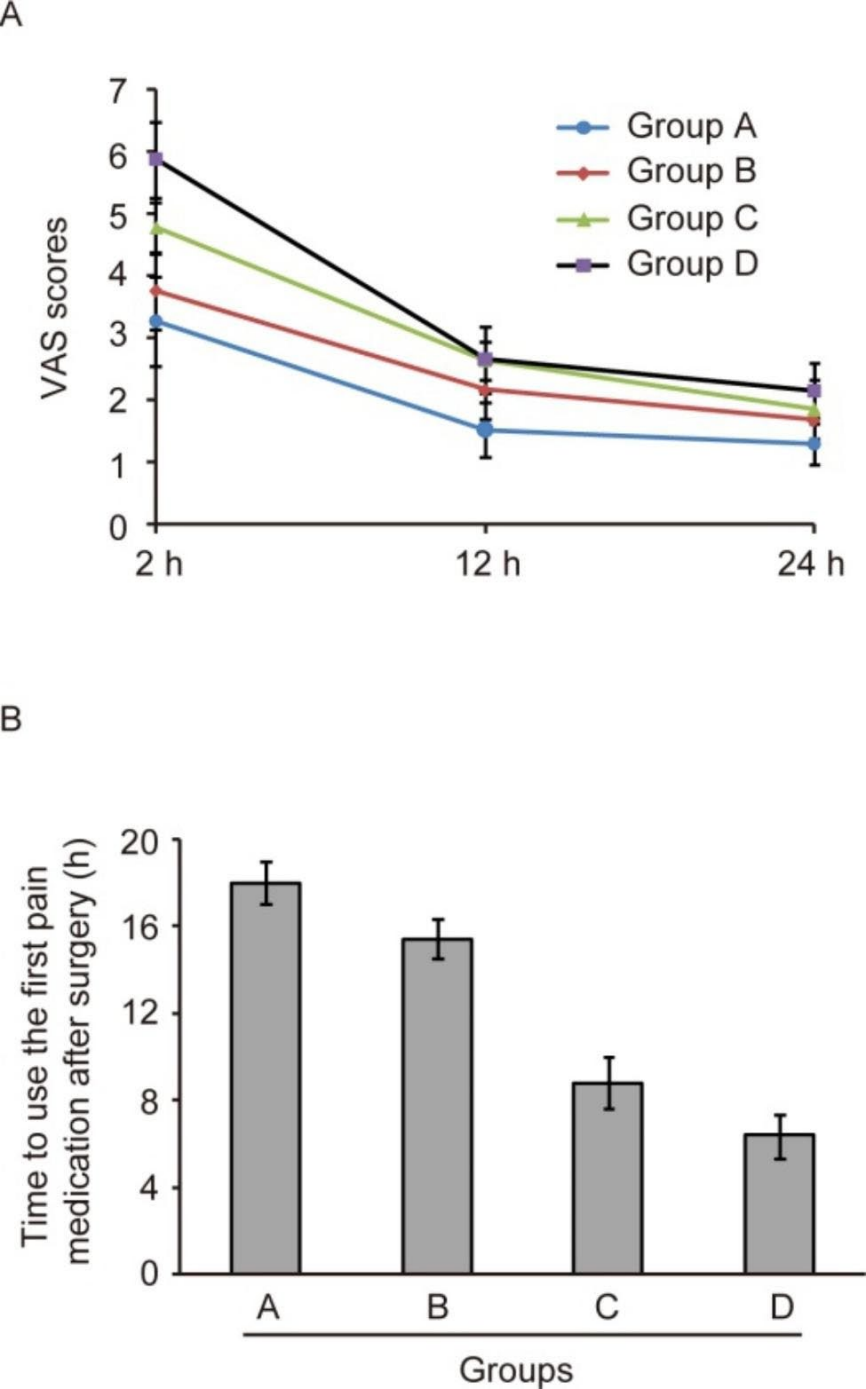
Comparison of VAS scores (**A**), and time to use the first pain medication (**B**) of breast cancer patients in the four groups after surgery

The VAS scores for analgesic evaluation at 2 h, 12 h, and 24 h after extubation were compared among the four groups of patients. As shown in Fig. 3A, there were significant differences of VAS scores at different time points after repeated-measures ANOVA (F = 929.712, *P* < 0.05). The VAS scores at 12 and 24 h were significantly lower than those at 2 h (*P* < 0.05). Furthermore, the four groups of breast cancer patients had differences in VAS scores (F = 153.199, *P* < 0.05). And the trends of VAS scores were also different among the four groups (F = 23.939, *P* < 0.05). VAS score results indicate that sufentanil preemptive analgesia combined with psychological intervention can decrease the pain of breast cancer patients after surgery.

The time to use the first pain medication after surgery in the four groups was compared by one-way ANOVA. As shown in Fig. 3B, the difference in time to use the first pain medication after surgery was statistically significant among the four groups (F = 608.792, *P* < 0.05). Further SNK test showed that group A had the longest time to use the first pain medication after surgery (*P* < 0.05), while patients in group D had the shortest time (all *P* < 0.05). However, there was no difference of the use of postoperative analgesics among the four groups (Table 1, *P* > 0.05). These results show that sufentanil preemptive analgesia combined with psychological intervention can extend the time to use the pain medication of breast cancer patients after surgery.

**Table 1:** Cases of the use of postoperative analgesics at different time points after extubation among the four groups [n(%)]

Groups	2 h	12 h	24 h
Group A	0 (0)	1 (3.57)	0 (0)
Group B	1 (3.57)	2 (7.14)	1 (3.57)
Group C	3 (10.71)	3 (10.71)	1 (3.57)
Group D	4 (14.28)	1 (3.57)	1 (3.57)

### Group comparison of adverse reactions

The adverse reactions of patients after surgery, such as vomiting, itching, dizziness, and restlessness were recorded and compared among the four groups. As shown in Table 2, there was no statistically significant difference in the incidence of above postoperative adverse reactions among the four groups (all *P* > 0.05). In addition, patients in the four groups had stable respiratory and circulatory functions after surgery, and no serious complications such as respiratory depression and gastrointestinal bleeding occurred.

**Table 2:** Cases of adverse reactions after surgery among the four groups [n(%)]

Groups	Vomiting	Itching	Dizziness	Restlessness
Group A	0 (0)	1 (3.57)	0 (0)	0 (0)
Group B	0 (0)	1 (3.57)	1 (3.57)	1 (3.57)
Group C	1 (3.57)	0 (0)	0 (0)	2 (7.14)
Group D	0 (0)	0 (0)	2 (7.14)	3 (10.71)

## Discussion

Although the surgery technology of breast cancer in China has developed rapidly in recent years, the surgery trauma increased by psychological stress is often neglected [23, 24]. Therefore, it is especially important to reduce the psychological stress response of patients before surgery. For breast cancer patients undergoing radical surgery, the treatment of postoperative pain needs to be considered from different perspectives [25], including psychological interventions before surgery [26]. When the surgeon clearly informs the breast cancer patient that surgical treatment is the optimal option for the current state of the disease, the patient has uncertainty and various anxieties about the development of the disease, which may cause excessive secretion of adrenaline [27].

Psychological intervention refers to the process of influencing the mental activities, personality traits or psychological problems of certain subjects in a planned and systematic manner under the guidance of psychological theories to make them change towards the desired goal [28]. Through PPST, breast cancer patients and their families can understand the knowledge about surgery and perioperative period, and reduce the fear brought by unfamiliar scenes. After the anesthesiologists instruct anesthesia and surgery precautions, cooperation points, and answer the questions raised by patients and their families, there will be a good communication relationship between patients and anesthesiologists. Moreover, breast cancer patients can relieve tension and fear before surgery, and enter the operating room at ease. During this process, anesthesiologists will get the general information of patients, such as weight, personality, anxiety and nervousness, etc., and provide targeted individual psychological interventions to address the problems from patients and give the effective psychological orientation and awareness in advance. Before surgery, the psychological interventions to patients provided by anesthesiologists can alleviate the fluctuations of the intrinsic vital signs in patient body brought by adverse emotions. The detailed preoperative explanation and communication will make patients prepared psychologically, which can improve their cooperation, effectively reduce the stress reactions generated during the perioperative period, shorten the anesthesia awakening time, and reduce the postoperative pain. Patients will also understand the anesthesia steps and postoperative recovery process in advance to reduce unnecessary excessive stress and make them emotionally stable [29].

In this study, we found that the use of psychological support interventions can encourage breast cancer patients to adjust their emotions and alleviate or even eliminate anxiety and depression of patients before surgery. Through the psychological support intervention, patients are shown to establish positive coping strategies and achieve a better preoperative psychological state, which then can reduce the postoperative pain to a certain extent [30].

Because postoperative pains often inhibit the wound healing and further aggravate the anxiety in patients, measures to prevent transmission to the central nervous system even before the body is exposed to noxious stimuli in such patients can eliminate or reduce postoperative pain to some extent. From the perspective of clinical effect, the analgesic effect of a single local nerve block is slightly poor, while the analgesic effect of continuous catheter nerve block is good. But it is difficult to carry out in the actual clinical work. Intravenous administration with preemptive analgesia is simple, fast, and effective. The use of sufentanil as preemptive analgesia can reduce the pain sensitivity in the central nervous system [31]. Sufentanil is a highly selective µ-opioid agonist and an N-4 derivative of fentanyl. It not only has a rapid onset of action, analgesic effect, and mild cardiovascular and respiratory depression, but also has no histamine release [32, 33]. With safe and mature clinical use, sufentanil is a cost-effective and a very good choice for advanced analgesic drugs [34]. It can reduce intraoperative agitation and postoperative pain and prevent pain from interfering with wound healing. Therefore, sufentanil preemptive analgesia combined with psychological support is an integrated management measures from human bio-psychology multi-perspectively and multi-dimensionally that can achieve more optimal results. In addition, we took the VAS scores at 2, 12 and 24 h after extubation, which were pain-sensitive time points, as the reference standard for postoperative pain judgment.

## Conclusions

Sufentanil preemptive analgesia combined with psychological support is an effective analgesia method for breast cancer patients undergoing radical surgery, which deserves to be promoted in clinic.

## Data Availability

The datasets used and/or analysed during the current study are available from the corresponding author on reasonable request.

## Abbreviations

PPST: Perioperative psychological support therapy
VAS: Visual analogue scale/score
